# The plant longevity gene *AHL15* delays leaf senescence by repressing *ORESARA1* and cytokinin degradation

**DOI:** 10.1111/tpj.71177

**Published:** 2026-10-14

**Authors:** Thalia Luden, Petra Amakorová, Ellora Basu, Ondřej Novák, Salma Balazadeh, Remko Offringa

**Affiliations:** ^1^ Plant Developmental Genetics Institute of Biology Leiden, Leiden University Sylviusweg 72 Leiden 2333 BE the Netherlands; ^2^ Laboratory of Growth Regulators, Faculty of Science Palacký University and Institute of Experimental Botany, The Czech Academy of Sciences CZ‐77900 Olomouc Czech Republic; ^3^ Plant Stress Biology, Institute of Biology Leiden Leiden University Sylviusweg 72 2333 BE Leiden the Netherlands; ^4^ Present address: Institute of Plant Sciences – Paris Saclay (IPS2) University of Paris‐Saclay Bat 630, Avenue des Sciences 91190 Gif‐sur‐Yvette France; ^5^ Present address: Albrecht‐Von‐Haller‐Institute for Plant Sciences University of Göttingen Julia‐Lermontowa‐Weg 3 D‐37077 Göttingen Germany

**Keywords:** AHL15, leaf senescence, ORESARA1, cytokinin, CKX

## Abstract

The *Arabidopsis thaliana* (Arabidopsis) *AT‐HOOK MOTIF NUCLEAR LOCALIZED 15 (AHL15)* gene is associated with various longevity phenotypes and extends the life span of plants when overexpressed. In this study, we show that, in addition to previously described longevity phenotypes, constitutive overexpression of *AHL15* in Arabidopsis delays leaf senescence, whereas *ahl15* loss of function accelerates this process. Dexamethasone‐induced nuclear localization of AHL15‐GR during dark‐triggered senescence results in a stay‐green phenotype and represses the expression of several early senescence‐associated genes. Among these, the *ORESARA1 (ORE1)* locus is directly bound by AHL15, suggesting a direct delaying effect of AHL15 on senescence. Furthermore, we demonstrate that AHL15 acts by directly repressing the expression of several *CYTOKININ OXIDASE (CKX)* genes involved in cytokinin inactivation, resulting in delayed degradation of cytokinins during dark‐induced senescence. Cytokinins are known to inhibit senescence, and this, together with the downregulation of *ORE1* expression, explains the delaying effect of AHL15 on senescence.

## Significance Statement

Here, we show that, in addition to its previously reported effects on plant aging processes, the *AT‐HOOK MOTIF NUCLEAR LOCALIZED*‐family protein AHL15 also delays leaf senescence, revealing a previously unrecognized role in leaf (organ) longevity. Our results demonstrate that the senescence program is delayed by AHL15 by two parallel pathways, resulting in the strong stay‐green phenotype of *AHL15* overexpressing plants. First, AHL15 directly represses transcription of *ORESARA1,* a master regulator of senescence. Second, AHL15 represses expression of *CYTOKININ OXIDASE* genes, thereby delaying the breakdown of the senescence‐delaying hormone cytokinin. To illustrate this, we measured levels of different cytokinin types in senescent Arabidopsis leaves, providing the first detailed characterization of cytokinins during the senescence process.

## INTRODUCTION

Leaf senescence is the process during which the photosynthetic machinery of leaves is catabolized and recycled for use in other parts of the plant (Guo et al., 2021; Lim, Kim, & Nam, 2007; Schippers et al., 2015). It is the final developmental stage of the leaf, and its progression is tightly controlled by various transcription factors (TFs). Based on research primarily conducted in the model plant *Arabidopsis thaliana* (Arabidopsis), the TFs ORESARA1 (ORE1) and NAC‐LIKE, ACTIVATED BY AP3/PI (AtNAP), have been identified as major regulators of senescence (Guo & Gan, 2006; Kim et al., 2009; Qiu et al., 2015). Loss of function of *ore1*, *nap*, or other positive regulators of senescence results in a stay‐green phenotype (Guo & Gan, 2006; Kim et al., 2009), and ORE1 regulates the transcription of numerous genes in the senescence pathway (Balazadeh et al., 2010). Upon initiation of senescence, genes involved in chlorophyll degradation, also referred to as chlorophyll catabolic genes (CGGs), are upregulated by ORE1, NAP, and other TFs in the senescence pathway, and together, the resulting enzymes degrade chlorophyll (Chl) into smaller molecules that can be exported to other tissues in the plant, resulting in the characteristic yellowing of senescent leaves (Qiu et al., 2015; Woo et al., 2019; Yang et al., 2015). The remobilization of nutrients from older leaves to other parts of the plant helps regulate nutrient availability and distribution, thereby increasing the plant's chances of survival during periods of nutrient scarcity (Schippers et al., 2015). However, when senescence negatively impacts the overall quality of a crop, such as in leafy vegetables or in ornamental plants, delayed senescence can be a desirable trait. Therefore, improving the control of leaf senescence is essential for enhancing crop quality.

Leaf senescence can be induced by various factors, including leaf age, dark incubation, nutrient shortage, and the application of hormones such as ethylene, jasmonic acid, abscisic acid, salicylic acid, or strigolactones (Woo et al., 2019). In Arabidopsis, ethylene triggers a signaling cascade that activates the transcription factor ETHYLENE INSENSITIVE 3 (EIN3), which in turn promotes the transcription of *ORE1* and *NAP* (Kim et al., 2014; Li et al., 2013). Additionally, dark incubation induces transcription of both *ORE1* and *EIN3* via PHYTOCHROME INTERACTING FACTOR4 (PIF4) and PIF5 (Song et al., 2014). In monocarpic plants, flowering also triggers leaf senescence, allowing the relocation of nutrients from leaves to seeds and thereby enhancing the offspring's chances of survival (Thomas, 2013). For example, in Arabidopsis, the whole plant senesces within a few weeks after flowering. This process is controlled by several TFs, among which MYB2 and FRUITFULL (FUL), and coincides with reduced production of a class of plant hormones called cytokinins (CKs) (Balanzà et al., 2018; Guo & Gan, 2011).

In contrast to most other plant hormones that induce senescence, CKs inhibit leaf senescence: Exogenous CK application significantly delays leaf senescence in various plant species (Gan & Amasino, 1995; Hallmark & Rashotte, 2020; Pokorná et al., 2021; Richmond et al., 1957). The senescence‐delaying activity of CK is mediated by the CK receptor ARABIDOPSIS HISTIDINE KINASE 3 (AHK3), which phosphorylates ARABIDOPSIS RESPONSE REGULATOR 2 (ARR2) upon CK perception (Kim et al., 2006). ARR2 then promotes expression of *CYTOKININ RESPONSE FACTOR 6* (*CRF6*), a TF that negatively regulates leaf senescence via an unknown mechanism (Zwack et al., 2013). *ahk3* loss‐of‐function mutants show accelerated senescence phenotypes, and the senescence‐delaying effect of CK treatment is reduced in these plants because of their weakened ability to transduce CK signals (Kim et al., 2006). A similar phenotype can be seen in *crf6* mutants (Zwack et al., 2013).

CKs are synthesized predominantly by enzymes of the ISOPENTENYL TRANSFERASE (IPT) and LONELY GUY (LOG) families. IPT‐family enzymes are responsible for adding a prenyl group to N^6^ of ATP or ADP (Kakimoto, 2001; Takei et al., 2001). Additional enzymes such as CYP735A are involved in making changes to this prenyl group, thereby producing precursors to different CK variants (Argueso & Kieber, 2024). Biological activation of these CK precursors relies on the removal of the riboside group by LOG‐family enzymes, which are highly redundant and of which LOG7 and LOG8 appear to be the most important in Arabidopsis (Kuroha et al., 2009; Tokunaga et al., 2012). CK degradation is predominantly accomplished by enzymes of the CYTOKININ OXIDASE/DEHYDROGENASE (CKX) class. CKX enzymes irreversibly cleave off the N^6^ side chains from CKs, thereby inactivating them permanently (Galuszka et al., 2000; Schmülling et al., 2003). Knockout of the rice *ckx11* gene causes a delay in leaf senescence, indicating that reduced CK degradation negatively affects senescence (Zhang, Fang, et al., 2021). On the contrary, overexpression of *LOG4* delays leaf senescence in Arabidopsis (Kuroha et al., 2009) and causes pleiotropic effects. In addition, ectopic expression of the *Agrobacterium tumefaciens IPT* gene under the senescence‐specific *SAG12* promoter delays leaf senescence in several crops (Gan & Amasino, 1995; Jordi et al., 2000; McCabe et al., 2001). Thus, leaf senescence can be regulated by controlling the expression of the CK biosynthesis genes of the *IPT* and *LOG* families and by regulating the expression of the CK‐degrading *CKX* genes.

While CKs play an important role in senescence, the regulatory mechanisms that control CK‐mediated repression of senescence, as well as the CK species most active during the senescence program, remain elusive. So far, only two TFs that regulate CK levels during senescence have been identified: MYB2 and NAP (Guo & Gan, 2011; Hu et al., 2021; Li et al., 2023). In wild‐type plants, *IPT* expression in the leaf is silenced by MYB2, whose expression increases with progressing age (Guo & Gan, 2011). It was shown that *MYB2* expression in Arabidopsis and the legume *Caragana intermedia* can be repressed by proteins of the S40 family, and overexpression of *S40*‐family genes results in enhanced CK levels and a stay‐green phenotype (Yang et al., 2022). At the same time, *CKX3* expression is induced by the senescence‐promoting TF NAP, resulting in enhanced CK degradation (Hu et al., 2021). The repression of *CKX* expression by NAP has been observed in Arabidopsis and Chinese cabbage, indicating that this pathway is conserved (Hu et al., 2021; Li et al., 2023). These results show a clear link between CK and age‐induced senescence, but additional regulatory mechanisms that control the expression of CK biosynthesis and degradation genes during the senescence process remain to be identified.

Previously, we identified the Arabidopsis gene *AT‐HOOK MOTIF NUCLEAR LOCALIZED15 (AHL15)* as a regulator of whole plant longevity. Overexpression of *AHL15* converts monocarpic Arabidopsis into a polycarpic plant (flowering multiple times) by delaying flowering and axillary meristem maturation. This results in an extended life span and prolonged flowering period, accompanied by enhanced vegetative development from axillary meristems and increased branching and secondary growth, overall enhancing the biomass yield via increased leaf production and enhanced seed set. By contrast, *ahl15* loss‐of‐function or perturbation of *AHL* gene function accelerates axillary meristem maturation and reduces flowering time and whole plant longevity (Karami et al., 2020; Rahimi et al., 2022a). CK levels are increased in stems of *AHL15* overexpressing plants, explaining the enhanced branching and secondary growth in these plants (Rahimi et al., 2022b). In Arabidopsis, the *AHL* gene family comprises 29 members that are subdivided into two clades (clades A and B) (Zhao et al., 2014). Previous research has demonstrated that members of the clade‐A *AHL* subfamily, including *AHL15*, exhibit a high level of functional redundancy (Karami et al., 2020; Yun et al., 2012; Zhao et al., 2013). Lim, Kim, Breeze, et al. (2007) showed that overexpression of the clade‐A member *AHL27* delays leaf senescence and reprogram, whereas a recent study has identified the clade B member *AHL9* as a promoter of leaf senescence, suggesting that clade‐A and ‐B AHLs may function antagonistically (Lim, Kim, Breeze, et al., 2007; Zhou et al., 2022). However, the precise mechanism via which AHLs influence the senescence pathway is still poorly understood.

Here, we show that overexpression of *AHL15* strongly delays leaf senescence, while loss‐of‐function *ahl15* mutants exhibit slightly earlier senescence. Our results show that the expression of several senescence marker genes, including *ORESARA1* (*ORE1*), is repressed when AHL15 activity is induced and that AHL15 binds to the *ORE1* locus, indicating that AHL15 contributes to the repression of the senescence program. Additionally, we found that AHL15 binds to regions upstream of the *CKX2*, *CKX3*, and *CKX5* genes and that induction of AHL15 activity leads to nearly complete silencing of their expression. This silencing coincides with a slower decline in CK levels during dark‐induced senescence. Together, our data indicate that AHL15 delays leaf senescence through two parallel pathways: by repressing the expression of *ORE1* and by repressing *CKX* expression and their degradation of CK.

## RESULTS

### *AHL15* overexpression delays leaf senescence

Given that overexpression of *AHL15* in Arabidopsis extends the plant's life span (Karami et al., 2020), we were curious whether this gene could also enhance longevity at the organ level, specifically in leaves. Previously, delayed leaf senescence has been reported for *AHL27* overexpressing lines (Lim, Kim, Breeze, et al., 2007). Here, we tested whether a similar senescence phenotype could also be observed in *p35S:3xFLAG‐AHL15* plants (hereafter referred to as *AHL15* OX). To this end, we compared senescence in the fifth leaf of Col‐0 (wild type) and *ahl15* knockout plants with two independent *AHL15* OX lines in both dark‐induced (Figure 1) and age‐dependent conditions (Figure S1). To quantify senescence, we measured leaf chlorophyll (Chl) using two methods: (1) Chl extraction using acetone followed by spectrophotometry (Arnon, 1949) and (2) a recently developed colorimetric assay. This assay measures the pixel intensity value in Red, Green, and Blue channels of digital images, which can be used to calculate the normalized Red value (Rn; Red/(Red+Green+Blue)). Previously, we have demonstrated that the Rn value shows a significant negative correlation with the total Chl content of leaves, which makes it an appropriate non‐invasive method for Chl quantification (Luden et al., 2025).

**Figure 1:**
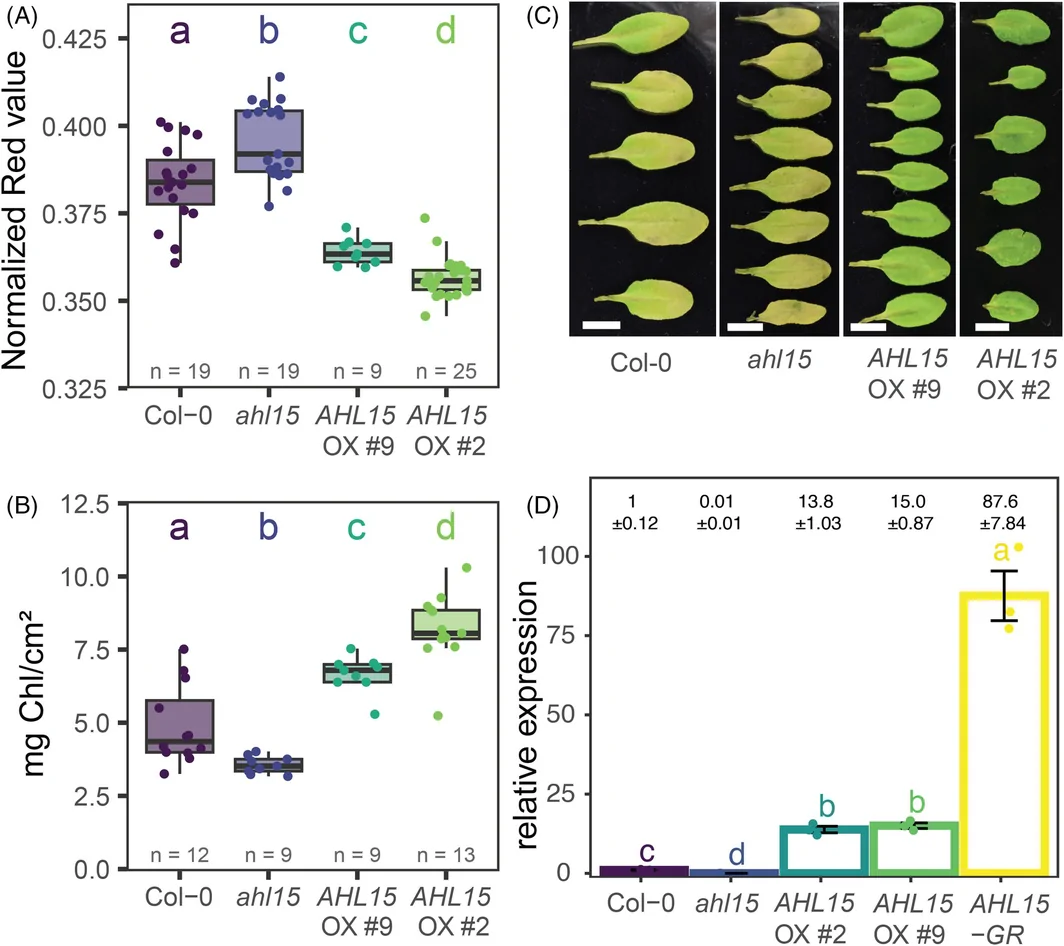
Dark‐induced leaf *s*enescence is delayed by *AHL15* overexpression and accelerated by *ahl15* loss‐of‐function. (A–C) The fifth leaf of 23‐day‐old wild‐type (Col‐0), *ahl15* mutant, or *p35S:3xFLAG‐AHL15* (*AHL15 OX*) Arabidopsis plants was detached and floated on 5 mL (5 mM) MES buffer for 5 days in the dark to induce the senescence process. Leaves were imaged for chlorophyll quantification as the normalized Red value (Rn) (A). Chlorophyll was extracted and quantified by spectrophotometer (B). Representative images of leaves of each genotype (C). Differences between genotypes in A and B were compared by a Kruskal–Wallis test, and significance groups are indicated by letters (*n* = 9–25; *P* < 0.05). White bars in C represent 1 cm. (D) Relative expression of *AHL15* in the fifth leaf of 23‐day‐old wild‐type (Col‐0), *ahl15* loss‐of‐function, *AHL15* overexpression (*AHL15 OX* lines 2 and 9), and *35S:AHL15‐GR* plants. The average relative expression level of each genotype, normalized to *ACTIN2* and Col‐0 using the ΔΔCT method, is given above the corresponding bar. Error bars show the standard error of the mean; groups were compared with the Kruskal–Wallis test, and different letters indicate significant differences between groups (*P* < 0.05).

In dark‐induced conditions, the senescence of the fifth leaf was significantly delayed in the *AHL15 OX* lines compared to wild type (Col‐0), as evidenced by a lower Rn value (Figure 1A,C) and higher total Chl content (Figure 1B,C). By contrast, *ahl15* loss‐of‐function plants showed a mild but significant increase in Rn value and a decrease in total Chl content compared to Col‐0. The reduced or enhanced leaf senescence in the *AHL15 OX* lines or *ahl15* loss‐of‐function plants, respectively, relative to Col‐0 correlated well with the *AHL15* expression levels (Figure 1D), demonstrating that *AHL15* delays leaf senescence under dark‐induced conditions.

Similarly, at 42 days after germination, the older leaves of wild‐type and *ahl15* knockout plants started to senesce, whereas leaves of the same age in *AHL15* OX lines stayed green, indicating that age‐dependent senescence could be delayed by *AHL15* overexpression (Figure S1A). To monitor this in more detail, we harvested the fifth leaves of different plants at 21, 24, 27, 30, 34, and 36 days of emergence and used images of these leaves to measure the Rn value (Figure S1B,C). This showed that the progression of senescence in the fifth leaf was slightly delayed in *AHL15* OX plants (most strongly in *AHL15* OX line 2) compared to wild‐type and *ahl15* loss‐of‐function plants, suggesting that AHL15 may play a role in age‐dependent senescence as well. The latter is in line with the expression of *AHL15*, which is on in young developing leaves and turned off when leaves are fully grown (Figure S2).

Overexpression of *AHL15* and other clade‐A *AHLs* generally delays flowering (Karami et al., 2020; Street et al., 2008; Xiao et al., 2009), and AHL15 has been shown to delay vegetative phase change (Rahimi et al., 2022a). In addition, *AHL15* is expressed primarily in juvenile, newly developing leaves, and is absent in older and adult‐stage leaves (Rahimi et al., 2022a). To evaluate the effects of *AHL15* overexpression on the leaf senescence phenotype independently of other developmental processes, we used a dexamethasone (DEX)‐inducible *p35S:AHL15‐GR* line that strongly expresses an *AHL15‐GR* fusion gene (Figure 1D) of which the encoded AHL15‐GR fusion protein translocates to the nucleus upon DEX treatment (Karami et al., 2020). During the dark incubation period, leaves were floated on 5 mL MES solution, to which DEX was added to a final concentration of 10 μM to induce AHL15 activity simultaneously with the senescence process. DEX‐treated *p35S:AHL15‐GR* leaves showed a similar delayed senescence phenotype as *AHL15 OX* leaves, whereas mock‐treated *p35S:AHL15‐GR* leaves senesced like wild‐type leaves (Figure 2A–C). Rn and chlorophyll measurements on mock and DEX‐treated *p35S:AHL15‐GR* leaves confirmed the phenotypic observations (Figure 2A,B).

**Figure 2:**
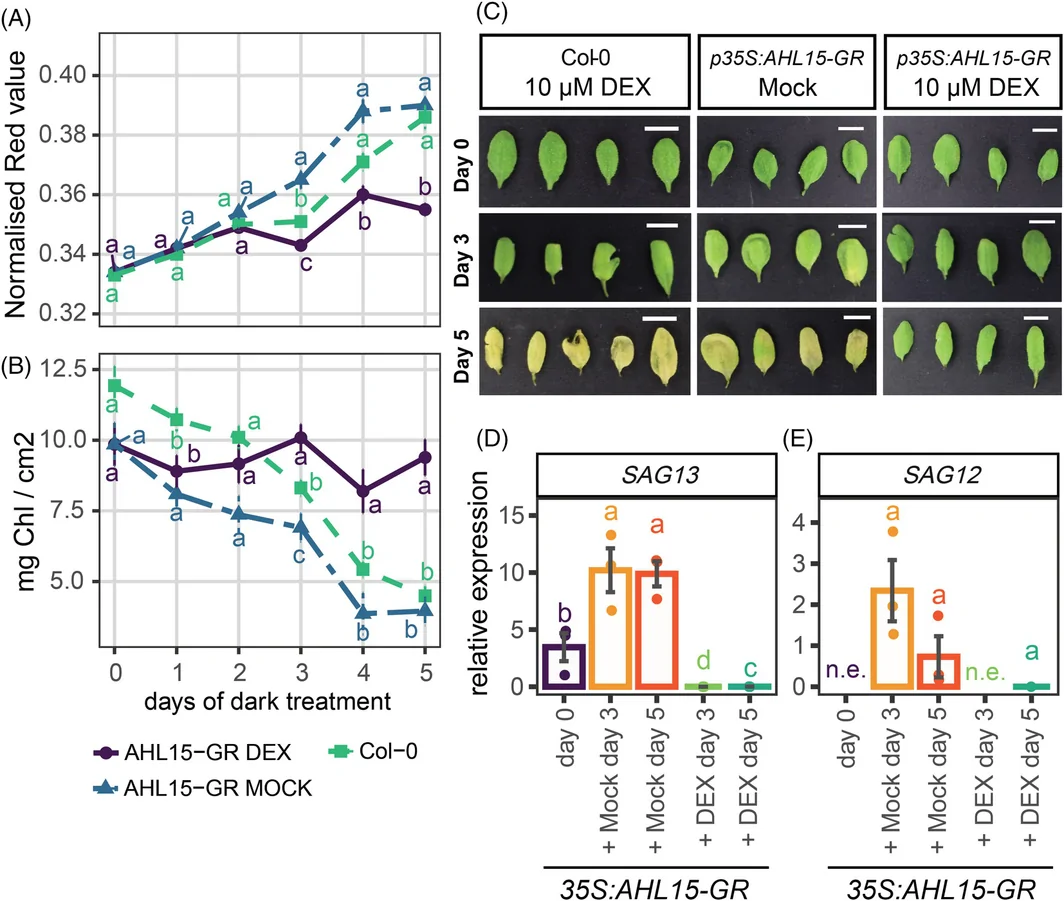
DEX‐induced nuclear localization of AHL15‐GR inhibits leaf senescence. (A–C) The fifth leaf was detached from 23‐day‐old wild‐type (Col‐0) or *p35S:AHL15‐GR* plants and incubated in the dark on 5 mM MES buffer with or without 10 μM DEX for the indicated number of days. The leaf chlorophyll content was quantified over 5 days of dark incubation by image‐based calculation of the normalized Red value (A) or by direct chlorophyll extraction (B) (*n* = 8 for days 1, 2, and 4, and *n* = 16 for days 0, 3, and 5). The quantification was repeated twice with similar results. Representative images of leaves from each group before and after 3 or 5 days of dark incubation (C). White bars in (C) represent 1 cm. (D, E) Expression of the senescence‐associated genes *SAG13* (D) and *SAG12* (E) in the DEX‐or Mock‐treated *p35S:AHL15‐GR* leaves shown in C, as determined by RT‐qPCR. RNA was extracted from three samples per time point and condition (*n* = 3), with each sample consisting of the fifth leaf of three individual plants. Expression was normalized to *ACTIN2*. *SAG12* expression could not be detected (n.e. for not expressed) at day 0 and in DEX‐treated leaves at day 3. Letters indicate significance groups calculated with the Kruskal–Wallis test (*P* < 0.05).

We next set out to characterize the pathway via which AHLs, and specifically AHL15, delay senescence. For this, we started by investigating the effect of DEX‐induced AHL15‐GR nuclear localization on senescence‐associated genes. Based on the Rn value and total Chl content of leaves incubated in the dark over 5 days, significant differences between DEX‐induced and non‐induced leaves (DEX‐treated *vs*. mock‐treated *p35S:AHL15‐GR*, or DEX‐treated Col‐0) became evident after 3 days of dark incubation. This indicates that senescence had been initiated at this time point in non‐induced leaves (Figure 2A–C). To determine whether AHL15 delays senescence at its onset or a later stage, we measured the expression of *SENESCENCE‐ASSOCIATED GENES (SAGs) SAG13* and *SAG12*, which are associated with early and late senescence, respectively (Lohman et al., 1994). RNA was isolated from leaves right before dark incubation and DEX treatment (day 0), and after 3 or 5 days of dark incubation with and without DEX treatment. A clear and significant difference in the expression of the early senescence marker *SAG13*, as well as the late senescence marker *SAG12*, was visible between DEX‐ and mock‐treated plants (Figure 2D,E), whereas the expression of *SEN1* was slightly but significantly reduced in DEX‐treated leaves after 5 days (Figure S3). As the expression of *SEN1* is induced by darkness (Chung et al., 1997; Oh et al., 1996), these results indicate that overexpression of *AHL15* predominantly affects the onset of senescence at an early stage and interferes with the response to darkness less strongly.

### AHL15 directly represses the expression of *ORE1* and several *CKX* genes

To further understand how AHL15 delays leaf senescence, we investigated whether AHL15 binds any senescence genes directly by consulting AHL15 ChIP‐seq data from *AHL15* overexpressing plants *(p35S:3xFLAG‐AHL15)* that we generated in a previous work (Luden et al., 2026). ChIP‐seq peaks of AHL15 were present up‐ and downstream of many genes, among which the *SQUAMOSA PROMOTER BINDING PROTEIN‐LIKE 9 (SPL9)* gene (Figure S4), whose expression was previously shown to be suppressed by AHL15 (Rahimi et al., 2022a). Interestingly, significant peaks were present at 600 bp upstream and 1 kb downstream of the senescence master regulator *ORE1*, which could explain the delayed senescence phenotype observed in *AHL15* overexpressing plants (Figure 3A). Therefore, we measured the expression of *ORE1* after senescence induction of DEX‐ or mock‐treated leaves and saw that its expression was strongly repressed in DEX‐treated leaves, implying a direct repression of this gene by AHL15 (Figure 3B).

**Figure 3:**
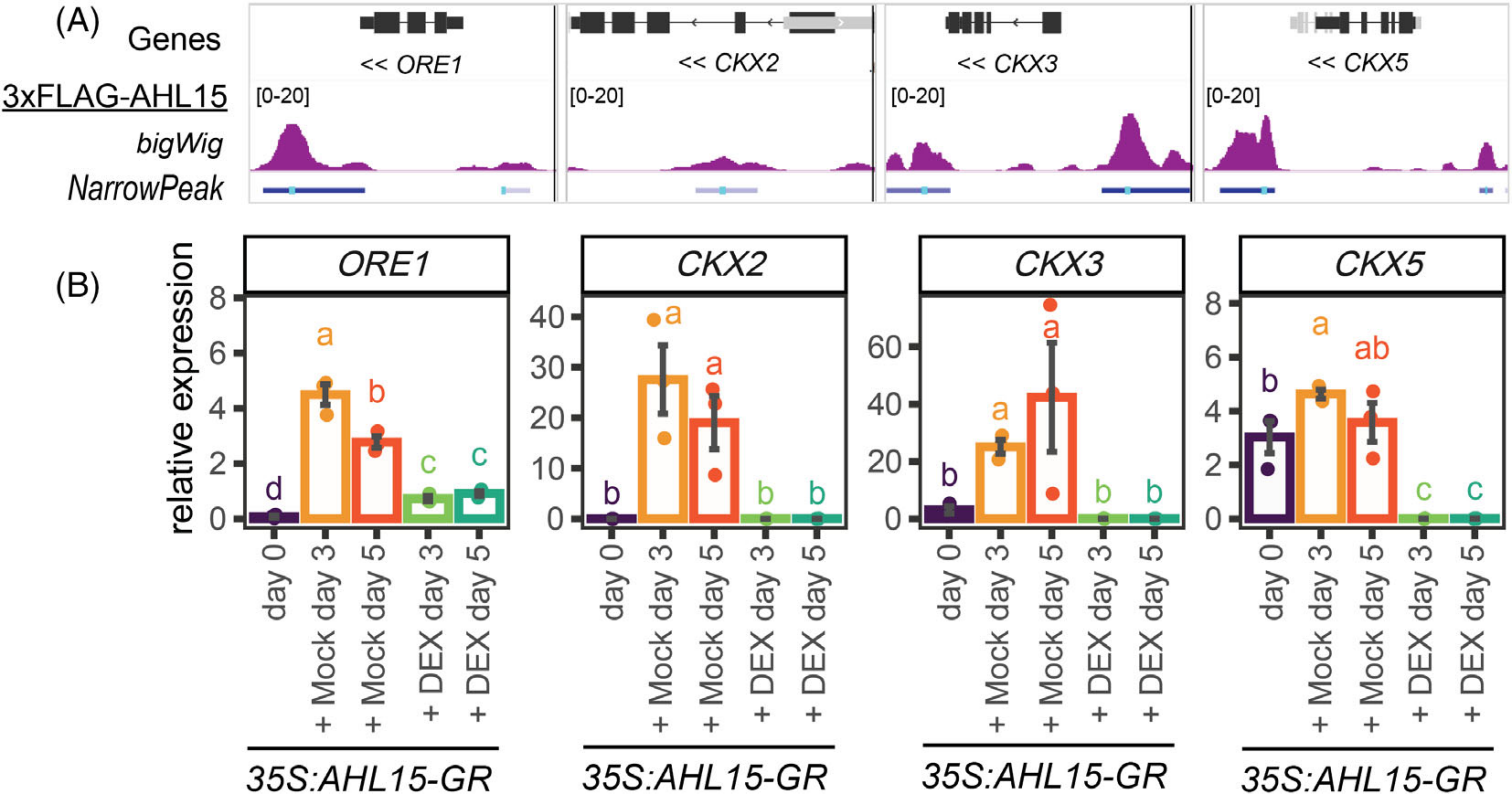
AHL15 binds to the *ORE1* and *CKX* loci to repress gene expression. (A) ChIP performed on the whole rosette of 31‐day‐old *p35S:3xFLAG‐AHL15* plants using an anti‐FLAG antibody. ChIP‐seq detected peaks of AHL15 binding 1 kb downstream and 600 bp upstream of *ORE1*, in the second intron of *CKX2*, 1.9 kb upstream and directly downstream of *CKX3*, and 2.3 kb upstream and 2 kb downstream of *CKX5*. Genes are shown in black (black blocks are exons, black lines are introns, gray blocks represent genes on the opposite strand), read alignments are shown in purple (bigwig track), and the location of significant peaks is indicated in blue, with a light blue bar indicating the peak summit (narrowPeak track). Similar results were obtained in three biological replicates. (B) The expression of *ORE1*, *CKX2, CKX3*, and *CKX5* is repressed by DEX‐induced AHL15‐GR activation in dark‐incubated leaves. Leaves of 23‐day‐old *p35S:AHL15‐GR* plants were floated on 5 mM MES buffer with or without 10 μM DEX for the indicated number of days in the dark. RNA used for RT‐qPCR was extracted from three samples per time point and condition (*n* = 3), with each sample consisting of the fifth leaf of three individual plants. Expression was normalized to *ACTIN2*. Different letters indicate significant differences between groups as calculated by the Kruskal–Wallis test (*P* < 0.05).

In addition, we identified the *CKX* genes *2*, *3*, and *5* (Figure 3A) and several CK biosynthesis genes (*IPT7*, *LOG1*, *LOG7*, *LOG8*; Figure S5A) among the significant ChIP‐seq peaks. Because of the previous finding that AHL15 promotes wood formation by enhancing CK levels (Rahimi et al., 2022b), and because CKs are known to have a delaying effect on leaf senescence (Guo & Gan, 2011; Hu et al., 2021; Kim et al., 2006; Yang et al., 2022), we hypothesized that the delayed senescence phenotype of *AHL15* overexpressing plants could in part be a result of increased CK levels. To test whether AHL15 regulates the expression of these genes, we measured the expression of each of these genes at 0, 3, or 5 days after senescence induction in DEX‐ or mock‐treated *p35S:AHL15‐GR* leaves. The expression of *LOG8* and the three *CKX* genes in mock‐treated leaves followed a similar pattern as the senescence markers *SAG13* and *SAG12*, and were all increased after 3 and 5 days of dark incubation (Figure 3B, Figure S5B). By contrast, the expression of *IPT7* and *LOG1* in mock‐treated leaves was lower than at day 0, and the expression of *LOG7* was not affected (Figure S5B). Interestingly, the expression of the *CKX* genes was significantly lower in DEX‐treated leaves compared to the mock treatment, implying that these genes are directly repressed by AHL15 (Figure 3B). The repression of *IPT7* expression upon dark incubation was delayed in DEX‐treated leaves, whereas the expression of *LOG1* and *LOG8* was repressed in comparison with mock‐treated leaves and *LOG7* expression was unaffected (Figure S5B). These data suggest that unlike in stem tissue, where AHL15 promotes CK biosynthesis (Rahimi, et al., 2022b), it delays leaf senescence rather by repressing the expression of cytokinin‐degrading CKX proteins. RT‐qPCR in the 5^th^ leaf of Col‐0, *ahl15*, and the two *AHL15* OX lines taken at 23 days (before senescence induction) showed that *CKX3*, *CKX5*, and *ORE1* were expressed at lower levels in *AHL15*‐overexpressing lines (Figure S6), indicating that their repression is independent of dark treatment. In addition, *CKX5* and *ORE1* were slightly, but not significantly, increased in *ahl15* plants compared to Col‐0 at this time point (Figure S6).

### Cytokinin levels are increased in *AHL15* overexpressing plants

The repression of *CKX* genes by AHL15 suggests that the inactivation of CKs is delayed in *AHL15‐*overexpressing leaves, resulting in increased CK levels that delay the onset of senescence. To test whether this is the case, we measured the levels of a wide range of CKs in dark‐induced *AHL15‐GR* leaves with and without DEX treatment before and during senescence (Figure 4; Figures S7–S12), providing a detailed characterization of CK during the senescence process. These measurements showed that total CK levels drop upon senescence induction in both DEX‐ and mock‐treated leaves, but that this decrease in CK levels is less pronounced in DEX‐treated leaves (Figure 4A). This trend was especially clear for the isopentenyladenine (iP)‐glucosides (Figure 4B; Figure S7), implying that they may be especially sensitive to degradation by CKX enzymes during the senescence process. Previous research has shown that both iP and iP‐9‐glucoside (iP9G), but not iP‐7‐glucoside (iP7G), can inhibit senescence in Arabidopsis cotyledons (Hallmark & Rashotte, 2020). Although iP in its base form was not present at detectable levels in any of the samples, the elevated levels of iP9G in DEX‐treated samples may partially explain the delayed senescence phenotype. In addition, the levels of trans‐zeatin base and trans‐zeatin Riboside (tZ and tZR) were reduced in all dark‐incubated samples, but their concentration remained significantly higher in DEX‐treated leaves (Figure 4C,D; Figure S8). Of all CK types, tZ‐base and tZR are among the most potent inhibitors of senescence (Hönig et al., 2018; Pokorná et al., 2021), and their elevated concentration in DEX‐ versus mock‐treated leaves could therefore be strongly involved in delaying senescence in DEX‐treated leaves.

**Figure 4:**
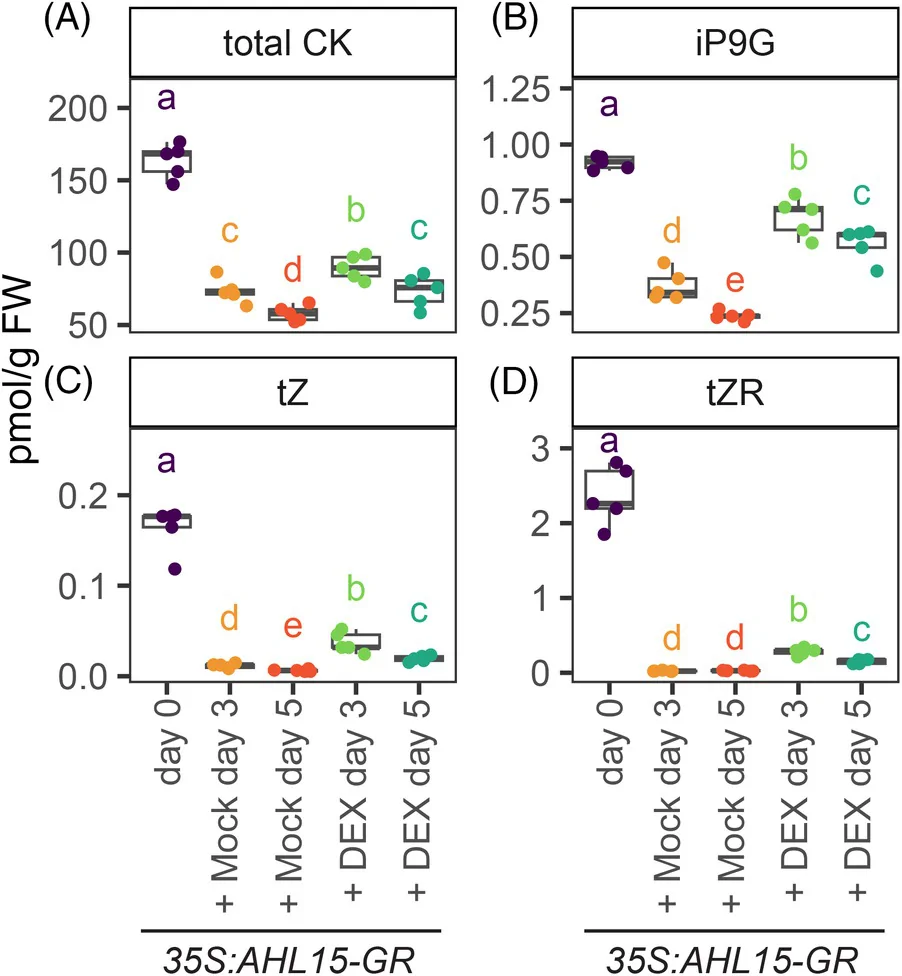
DEX‐induced AHL15‐GR activation delays the reduction in cytokinin (CK) levels in dark‐incubated leaves. (A–D) Quantification of the total CK (A), isopentenyladenine‐9‐glucoside (B), trans‐zeatin base (C), or trans‐zeatin riboside (D) level in the fifth leaf of 23‐day‐old *p35S:AHL15‐GR* plants floated on 5 mM MES with or without 10 μM DEX in the dark for the indicated number of days. For each sample, three leaves were pooled and five samples were measured for each condition (*n* = 5). Groups were compared using the Kruskal–Wallis test, and different letters indicate significant differences between groups (*P* < 0.05).

In addition to tZ and iP, we profiled the levels of cis‐zeatine (cZ; Figure S9) and dihydrozeatin (DHZ; Figure S10) during the senescence process. Interestingly, cZ‐base and cZG levels increased upon dark induction and were especially high after 5 days of DEX treatment (Figure S9). cZ has a weak effect on leaf senescence, and its function in the plant remains largely unknown (Gajdošová et al., 2011). Previous research has shown that DHZ levels drop during the senescence process in tobacco leaves (Hönig et al., 2018), and we observed a similar trend in our experiments (Figure S10). While DHZ‐base levels were undetectable, we found that DHZ derivatives were present at reduced levels in all samples and did not differ significantly between treatments, indicating that this form of CK is not affected by AHL15 or its downstream targets (Figure S10). Finally, we observed an overall increase in CK bases in DEX‐treated samples compared to untreated and mock‐treated samples (Figure S11), suggesting that other CK bases may also contribute to the AHL15‐mediated delay of senescence. The delayed senescence phenotype of DEX‐ vs mock‐treated leaves was verified before CK measurements (Figure S12).

Incubation of Col‐0 and *ahl15* leaves with tZ resulted in a significantly higher Chl content and a significantly lower Rn value (indicative of reduced Chl breakdown) compared to mock‐treated leaves (Figure S13), confirming that reduced CK levels are at least partially responsible for the dark‐induced leaf senescence. The Chl content did not significantly differ between Col‐0 and *ahl15* when measured by extraction, whereas the Rn value of both mock and tZ‐treated *ahl15* leaves was significantly higher than that of Col‐0 leaves. The latter is in line with the absence of *AHL15* expression in *ahl15* leaves (Figure 1D), resulting in a stronger senescence response upon dark treatment and a weaker delay of this response by CK treatment. To provide evidence that the reduced CK levels in dark‐incubated leaves are caused by CKX activity, we incubated Col‐0 and *ahl15* leaves with three established inhibitors of CKX activity (Nisler et al., 2024). CKX inhibitor treatment significantly delayed senescence in both *ahl15* and Col‐0 leaves (Figure S13), and even though this delay was less pronounced than with tZ treatment, the results indicate the involvement of CKX activity in reducing CK levels in dark‐incubated leaves. The fact that the senescence‐delaying effect of CK is less pronounced in *ahl15* compared to wild type is probably due to a weaker control over *ORE1* expression in the loss‐of‐function mutant background (Figure S6).

Taken together, our results show that AHL15 delays senescence through multiple mechanisms: by directly repressing *ORE1* expression and by downregulating *CKX* genes, thereby delaying the inactivation of CKs in leaves. The latter process results in elevated CK levels in *AHL15*‐overexpressing leaves, ultimately contributing to the delay in leaf senescence (Figure 5).

**Figure 5:**
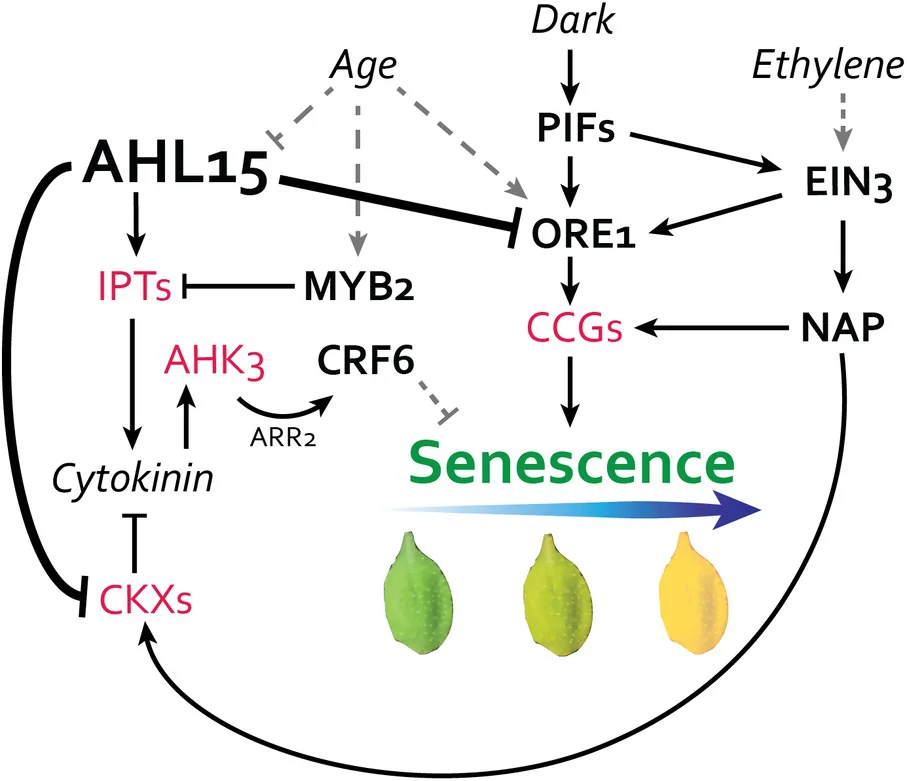
Model of AHL15‐mediated repression of senescence. AHL15 represses ORESARA1 (ORE1), which is a key positive regulator of the senescence transcriptional network. ORE1 can be induced by dark incubation via the PHYTOCHROME INTERACTING FACTORS (PIFs), by ethylene via ETHYLENE INSENSITIVE 3 (EIN3), or by age. ORE1 activates several downstream targets, among which are chlorophyll catabolic genes (*CCG*s), ultimately leading to chlorophyll catabolism and causing the characteristic yellowing of senescent leaves. The NAC‐LIKE, ACTIVATED BY AP3/PI (NAP) transcription factor acts in parallel to ORE1 and activates the expression of *CCG*s as well as the *CYTOKININ OXIDASE/DEHYDROGENASE* (*CKX*) genes, leading to the irreversible inactivation of cytokinins (CKs). CKs activate ARABIDOPSIS HISTIDINE KINASE 3 (AHK3), which represses leaf senescence via CYTOKININ RESPONSE FACTOR 6 (CRF6). CKs are produced by ISOPENTENYL TRANSFERASE (IPT) enzymes, which are transcriptionally repressed by MYB2 in aging plants. AHL15 represses *CKX* expression and promotes the expression of *IPT7*. Lower CKX levels result in slower CK degradation, resulting in higher CK levels and delayed leaf senescence. TFs are indicated in bold, enzymes in red, and hormones and external abiotic factors in italics.

## DISCUSSION

AHL15 has previously been shown to enhance plant longevity by delaying axillary meristem maturation, keeping these meristems in the vegetative state, and allowing plants to survive and grow again after each flowering cycle. This involves preventing stem cells in these meristems from transitioning from a vegetative to a reproductive state, resulting in delayed flowering and formation of aerial rosettes, thus allowing the plant to live longer (Karami et al., 2020; Rahimi et al., 2022a).

Here, we show that in addition to increasing the plant's life span, AHL15 also increases longevity at the organ (leaf) level. AHL15 delays leaf senescence by repressing the expression of *ORE1*, which encodes a key senescence regulator, and by inhibiting CK degradation through repression of *CKX* gene expression. These two processes are likely to act in parallel and together explain the strong stay‐green phenotype observed in *AHL15* overexpressing plants (Figure 5). Moreover, our data suggest that AHL15 acts predominantly by repressing gene expression, as observed for *ORE1* and three *CKX* genes.

ORE1 acts as a key transcriptional regulator of senescence, and its expression is induced by several senescence‐promoting environmental cues. Ethylene promotes the expression of *ORE1* and *NAP* via EIN3 (Kim et al., 2014; Li et al., 2013), ABA promotes *ORE1* expression via the TF ACTIVATING FACTOR1 (ATAF1) (Garapati et al., 2015), and dark incubation leads to enhanced *ORE1* transcription via the PIF TFs (Song et al., 2014). It was also shown that *ORE1* expression is post‐transcriptionally silenced in young leaves by miR164 and that this silencing is relieved with progressing age (Kim et al., 2009; Li et al., 2013). Here, we have identified AHL15 as a novel repressor of *ORE1* expression, suggesting that AHL15 can antagonize the ethylene‐ and dark‐induced senescence processes mediated by ORE1. In addition, *AHL15* is strongly expressed in juvenile plants and developing leaves and is gradually silenced over developmental age (Rahimi et al., 2022a). It is therefore possible that AHL15 can act as an additional age‐dependent repressor of *ORE1* expression in parallel to miR164 (Figure 5).

CK has long been recognized as a repressor of leaf senescence, yet its precise role and position within the senescence regulatory network remain relatively poorly understood. Application or overproduction of CK can delay the onset of leaf senescence (Argueso & Kieber, 2024; Gan & Amasino, 1995; Kim et al., 2006; Richmond et al., 1957), and it has been shown that CK degradation is promoted during the senescence process by NAP‐mediated upregulation of *CKX* expression (Hu et al., 2021; Li et al., 2023). MYB2 was shown to lower CK production in an age‐dependent manner by repressing the expression of several *IPT* genes, which are the rate‐limiting enzymes in the CK biosynthesis pathway (Guo & Gan, 2011). Our data show that AHL15 acts antagonistically to NAP in *CKX* transcriptional regulation and delays senescence by impairing CK degradation. In addition, we found that *IPT7* expression decreases in response to dark‐induced senescence and that this process is delayed after activation of AHL15‐GR by DEX treatment. Taken together, our results indicate that AHL15 acts as a positive regulator of CK in the senescence pathway and thereby acts antagonistically to NAP and MYB2 in this process (Figure 5).

Our experiments show that the expression of the CK biosynthesis genes *LOG1* and *IPT7* is downregulated upon senescence induction by dark treatment. On the contrary, we observed an increased expression of *LOG8* after senescence induction, and that *LOG7* expression is stable throughout the senescence process. In addition, the levels of cZ in its base form and most of its derivatives were increased in senescent leaves. Together, these data show that CK biosynthesis is not completely repressed but partially maintained during the senescence developmental program. Our data also confirm the previously reported induction of *CKX* expression during senescence (Hu et al., 2021; Li et al., 2023). This indicates that CK levels are regulated both by biosynthesis and by degradation during the senescence process and suggests a role for some CK variants in the senescence process. Finally, our data show that reduced breakdown of CK by direct silencing of *CKX* genes partially explains the delayed senescence phenotype of *AHL15* overexpressing plants. Previously, it was shown that the expression of *LOG4*, *IPT3*, and *IPT7* was decreased in *ahl15* knockout plants, resulting in lower CK levels and a decrease in radial xylem width (Rahimi et al., 2022b). Here, we observed delayed downregulation of *IPT7* in dark‐incubated leaves following DEX‐induced nuclear localization of AHL15‐GR, indicating that AHL15 targets are partially shared between stem and leaf tissue.

The leaf senescence phenotype of *ahl15* loss‐of‐function mutants is relatively mild (Figures S2, S13), suggesting that either *AHL15* itself plays a minor role in leaf senescence, or that other *AHL* genes take over its role in the mutant plants. *AHL* genes are highly functionally redundant, and developmental phenotypes are observed only in higher‐order *ahl* mutants (Karami et al., 2020; Rahimi, et al., 2022a; Xiao et al., 2009; Zhao et al., 2013). In addition, *AHL15* is expressed primarily in young juvenile leaves as its expression in adult leaves is repressed by SPL TFs (Rahimi et al., 2022a). In fact, no expression is observed in fully expanded juvenile leaves or in adult leaves of plants expressing the *pAHL15:GUS* reporter (Figure S2). It is therefore likely that higher‐order *ahl* mutants, especially of the clade‐A *AHL* genes that are expressed in adult leaves, show a stronger leaf senescence phenotype compared to the *ahl15* single mutant.

The functional redundancy among AHL proteins suggests that overexpression of other clade‐A *AHLs* could have similar effects as AHL15 on the expression of *ORE1* and *CKX* genes and the overall CK levels in the plant, and that reduced CK degradation is a general effect of high AHL dosage. Overexpression of at least one other clade‐A *AHL, AHL27*, has been shown to delay senescence (Lim, Kim, Breeze, et al., 2007), indicating that, like flowering time, the senescence‐delaying phenotype could be induced by multiple AHLs in this clade. It would therefore be interesting to see whether overexpression of other clade‐A *AHLs* has a similar senescence phenotype as *AHL15* and *AHL27*, and whether this coincides with increased CK levels in the leaves. Arabidopsis contains 15 clade‐A *AHLs*, and the genomes of most crop species contain a similar or higher number of *AHL* genes (Machaj & Grzebelus, 2021; Wang et al., 2023; Zhang, Peng, et al., 2021; Zhao et al., 2014; Zhao et al., 2020). Breeding efforts to improve plant longevity and shelf life could focus on enhancing the expression of clade‐A *AHLs*, which, due to their functional overlap and large number, represent promising targets for crop improvement.

## MATERIALS AND METHODS

### Plant material and growth conditions

Seeds were sown on damp soil (90% turf soil with 10% sand) and stratified for 3 days at 4°C before transfer to a climate chamber set at 21°C with a long day (16 h photoperiod) and 65% relative humidity. Seedlings were transferred to individual pots 1 week after sowing, and plants were watered weekly.

The *p35S:AHL15‐GR* overexpression and *ahl15* loss‐of‐function lines have been described previously (Karami et al., 2020). *p35S:3xFLAG‐AHL15* overexpression lines were generated by replacing the *AHL15‐GR* construct in the *p35S:AHL15‐GR* vector made by Karami et al. (2020) with a synthetic *3xFLAG‐AHL15* construct, which was transformed to *Agrobacterium tumefaciens* strain AGL1 by electroporation (den Dulk‐Ras & Hooykaas, 1995). Transgenic *p35S:AHL15* Arabidopsis Col‐0 lines were obtained by the floral dip method (Clough & Bent, 1998) and by subsequently selecting T1 and T2 plants on ½ MS medium supplemented with 15 mg/L phosphinotricin. Single locus transgenic lines, showing a 3:1 segregation ratio of resistant:susceptible plants in the T2 generation, were selected, and T3 seeds from homozygous T2 plants were subsequently used for senescence experiments.

For dark‐induced senescence assays, fifth leaves were detached from the plants at 23 days after the start of germination and floated on ca 5 mL 5 mM MES buffer (pH 5.6) in 5 cm ø Petri dishes, with a maximum of 10 leaves per dish. Petri dishes were sealed with parafilm, wrapped in aluminum foil, and stored in a cardboard box which was placed in the growth chamber to ensure a constant temperature of 20°C. For DEX treatments, a 10 mM dexamethasone (Thermo Fisher, Vilnius, Lithuania) stock solution dissolved in DMSO was diluted 1000‐fold in 5 mM MES buffer to a final concentration of 10 μM. For mock treatment, DMSO was diluted 1000‐fold in 5 mM MES buffer. For trans‐zeatin (Z0876; Sigma‐Aldrich, Schnelldorf, Germany) and CKX inhibitor treatments (shared by Jaroslav Nisler; (Nisler et al., 2024)), the respective compounds were diluted to 1 μM in 5 mM MES buffer from stock solutions, and an equivalent volume of solvent (DMSO) was added to 5 mM MES for mock treatments.

### Senescence measurements

For colorimetric measurements, the leaves were imaged on a dark background with a white reference card under uniform lighting with a Nikon DC3000 camera, with the following settings: ISO 160, exposure time 1/160 s. Image analysis was done in ImageJ with a custom plugin that measures Chl using RGB images (Luden et al., 2025). The analysis consisted of the following steps: white balancing of images based on a white reference sheet included in each picture; removal of background, conversion to an RGB image stack, automatic selection of individual leaves, and measurement of pixel intensity for each leaf in each of the red, green, and blue channels. The mean red, green, and blue values for each leaf were used to calculate the normalized red value (Rn; Red/(Red+Green+Blue)), which we previously showed correlates best with absolute Chl content (Luden et al., 2025).

After imaging for colorimetric measurements, two 5 mm ø leaf disks were taken from each leaf and stored at −80°C. Acetone‐based Chl extraction was done following the method described by Arnon (1949). Briefly, frozen leaf material was pulverized with a metal bead and dissolved in 200 μL 25 mM sodium phosphate buffer (pH 7) and 800 μL 80% acetone. Samples were then incubated at room temperature for 1 h with gentle shaking and centrifuged for 10 min at 2500 ***g***. 200 μL supernatant was then transferred to a clear‐bottomed 96‐well plate, and the absorption (D) at 645 and 663 nm was measured with a TECAN Spark plate reader (Männedorf, Switzerland). The total Chl content in mg/L was then calculated with the following formula: Chl_Total_ = 20.2 × D_645_ + 8.02 × D_663_, as described by (Arnon, 1949), and then corrected for the total area of leaf tissue used for Chl extraction. Spectrophotometry results and colorimetric results from ImageJ were processed in R (R Core Team, 2023). Plots were generated using ggplot2 (Wickham, 2016) and ggpubr (Kassambara, 2023), and genotypes or treatments were compared with the Kruskal–Wallis test by using the package Agricolae (de Mendiburu, 2023).

### ChIP‐seq of *AHL15*

AHL15 binding to genes in the senescence‐ and CK‐related pathways was identified by consulting ChIP‐seq data from whole 31‐day‐old *35S:3xFLAG‐AHL15* plants generated in a previous study (Luden et al., 2026). Figures 2A, S4, and S5A show the overlaid bigWig tracks of three *35s:3xFLAG‐AHL15* ChIP‐seq replicates, normalized to input.

### RNA isolation and RT‐qPCR

After imaging for colorimetric measurements, leaves were cut in half, of which one half was used for RNA extraction, while the other half of each leaf was used for Chl extraction. For each RNA extraction, material from three plants was pooled and snap‐frozen in liquid nitrogen, and tissue was pulverized with a metal bead using the Qiagen Tissuelyser. RNA was isolated with Trizol© reagent (Thermo Fisher Scientific, Vilnius, Lithuania) by following the standard protocol for Trizol‐based RNA extraction, with an additional ethanol wash step at the RNA precipitation stage. Next, 1 μg of RNA was aliquoted to a new tube and treated with DNase I (Thermo Fisher Scientific, Vilnius, Lithuania) at 37°C for 30 mins and used for cDNA synthesis with the RevertAid First Strand cDNA Synthesis Kit (Thermo Fisher Scientific, Vilnius, Lithuania) according to the kit's instructions. RT‐qPCR was performed with TB green ex‐Taq II (Takara Bio Europe, Saint‐Germain‐en‐Laye, France) on the QuantStudio 5 Real‐Time PCR machine (Applied Biosystems, Foster City, USA) in a reaction volume of 5 μL. Primers used for RT‐qPCR (Table S1) were synthesized by Sigma Aldrich (Haverhill, UK). Gene expression was normalized to *ACTIN2* expression by using the 2^‐ΔΔCT^ method (Livak & Schmittgen, 2001), and groups were compared with the Kruskal–Wallis test in R as described above.

### Cytokinin measurements

Quantification of cytokinin metabolites was performed on biological replicates according to the method described by Svačinová et al. (2012), including modifications described by Antoniadi et al. (2015). Samples (10 mg fresh weight) were homogenized and extracted in 1 mL of modified Bieleski buffer (60% methanol, 10% formic acid, and 30% water) together with a cocktail of stable isotope‐labeled internal standards (0.2 pmol of CK bases, ribosides, N‐glucosides, and 0.5 pmol of CK O‐glucosides, nucleotides per sample added). The extracts were applied onto an Oasis MCX column (30 mg/1 mL, Waters) conditioned with 1 mL each of 100% methanol and water, equilibrated sequentially with 1 mL of 50% (v/v) nitric acid, 1 mL of water, and 1 mL of 1 M formic acid, and washed with 1 mL of 1 M formic acid and 1 mL 100% methanol. Analytes were then eluted by two‐step elution using 1 mL of 0.35 M ammonium hydroxide aqueous solution and 2 mL of 0.35 M NH4OH in 60% (v/v) methanol solution. The eluates were then evaporated to dryness in vacuo and stored at −20°C. Cytokinin levels were determined by an ultra‐high‐performance liquid chromatography–electrospray tandem mass spectrometry (UHPLC–MS/MS) using stable isotope‐labeled internal standards as a reference (Rittenberg & Foster, 1940). Separation was performed on an Acquity UPLC® i‐Class System (Waters, Milford, MA, USA) equipped with an Acquity UPLC BEH Shield RP18 column (150 × 2.1 mm, 1.7 μm; Waters), and the effluent was introduced into the electrospray ion source of a triple quadrupole mass spectrometer Xevo™ TQ‐S MS (Waters, Milford, MA, USA). Five independent biological replicates, each consisting of material from the fifth leaf of three plants, were performed, including two technical replicates of each.

## Author Contributions

The project was conceived by TL and RO with input from SB. All experiments were performed by TL, except for the CK measurements performed by PA and ON, and the RT‐qPCR (Figure 1D; Figure S6) and CKX inhibitor (Figure S13) experiments performed by EB. https://doi.org/10.7554/eLife.109829.2 Figures were prepared by TL. The manuscript was written by TL and RO with input from SB, and the manuscript was read and approved by all authors.

## Conflict of Interest

The authors declare no competing financial interests or personal relationships that could have appeared to influence the work presented in this paper.

## Permission to Reproduce Material From Other Sources

The ChIP‐seq data presented here is sourced from: Luden et al. (2026). The authors give their permission to present part of their ChIP‐seq data in Figures 3A, S4, and S5A of this manuscript.

## Supporting information


**Figure S1.** AHL15 delays age‐dependent rosette leaf senescence.
**Figure S2.** *AHL15* is expressed in young, developing juvenile leaves.
**Figure S3.** AHL15 only mildly reduces dark‐induced *SEN1* expression in leaves.
**Figure S4.** AHL15 binds upstream of the *SPL9* gene.
**Figure S5.** AHL15 binds to cytokinin biosynthesis genes to differentially regulate their expression.
**Figure S6.** Expression of *CKX2, 3, 5*, and *ORE1* in the 5th leaf of 23‐day‐old Col‐0, *ahl15, AHL15* OX 2, and *AHL15* OX 9 plants before dark incubation.
**Figure S7.** AHL15 delays the dark‐induced decline in active CK levels in leaves.
**Figure S8.** AHL15 delays the dark‐induced decline in iP‐type CK levels in leaves.
**Figure S9.** AHL15 delays the dark‐induced decline in tZ‐ and tZR‐type CK levels in leaves.
**Figure S10.** The effect of AHL15 on cZ‐type CK levels in dark‐incubated leaves.
**Figure S11.** The effect of AHL15 on DHZ‐type CK levels in dark‐incubated leaves.
**Figure S12.** AHL15 preserves the chlorophyll content in leaves during dark incubation.
**Figure S13.** Trans‐zeatin or CKX inhibitor treatment decreases dark‐induced senescence in Col‐0 and *ahl15* loss‐of‐function leaves.
**Table S1.** RT‐qPCR primers used in this study.

## Data Availability

Raw data and materials are available upon request to RO.
