# Supplementary material for: The plant longevity gene AHL15 delays leaf senescence by repressing ORESARA1 and cytokinin degradation
Source: Plant J. 2026 Oct 14;128(1):e71177. doi: 10.1111/tpj.71177 (PMC13669646; doi:10.1111/tpj.71177)
Supplement: Supplementary file 1 [file TPJ-128-0-s001.docx]

**Supplementary figures**

**Figure S1:** AHL15 delays age-dependent rosette leaf senescence. **A:** Images of 42-day-old wild-type (Col-0), *ahl15*, and *AHL15 OX* (*p35S:3xFLAG-AHL15*) plants grown in 5 cm-wide pots under long-day conditions. **B:** Progression of senescence in the fifth leaf of wild-type (Col-0), *ahl15*, and *AHL15 OX* (*p35S:3xFLAG-AHL15*) plants at different days after emergence. White bars represent 1 cm. **C:** Chlorophyll content was quantified based on the images in panel **B** as the normalized Red value (Rn), with four leaves per time point (only three leaves are shown in **B** to preserve space). Different letters indicate significant differences between groups as calculated by the Kruskal-Wallis test (p < 0.05). Similar results were obtained in four independent experiments in different growth chambers.

**Figure S2:** *AHL15* is expressed in young, developing juvenile leaves. **A, B:** Images of 14-day-old (**A**) or 21-day-old (**B**) *pAHL15:GUS* plants grown in long-day (16h photoperiod) conditions and stained for GUS activity. The leaf numbers (in order of appearance) are indicated in white; cotyledons are labelled “c”. The size bar represents 5 mm.

**Figure S3.** AHL15 mildly reduces dark-induced SEN1 expression in leaves. Fifth leaves were detached from 23-day-old *p35S:AHL15-GR* plants and incubated in the dark on 5 mM MES buffer with or without 10 µM DEX for the indicated number of days. Expression was determined by qRT-PCR in three biological replicates (n=3), each composed of leaf material of three plants, using ACTIN2 as a reference. The Kruskal-Wallis test was used to compare conditions, and significan*t* differences between groups are indicated by different letters (p < 0.05).

**Figure S4:** AHL15 binds upstream of the *SPL9* gene. ChIP-seq was performed on chromatin isolated from 31-day-old *p35S:3xFLAG-AHL15* plants using an anti-FLAG antibody for immunoprecipitation. The *SPL9* gene is shown in black (blocks represent exons, black lines represent introns; transcription direction is indicated by a double arrow head), ChIP-seq read alignments from the samples are shown in purple (bigWig track), and significant peaks are shown in blue with a light blue bar indicating the peak summit (narrowPeak track) at approximately 500 bp upstream of the transcription start.

**Figure S5.** AHL15 binds to cytokinin biosynthesis genes to differentially regulate their expression. **A:** ChIP-seq was performed on chromatin isolated from 31-day-old *p35S:3xFLAG-AHL15* plants using an anti-FLAG antibody for IP. Genes are indicated black (black blocks are exons and black lines are introns), read alignments in purple (bigWig track), and the location of significant peaks in blue*,* with a light blue bar indicating the peak summit (narrowPeak track) at 1.4 kb and 3 kb upstream and in the 3’UTR of *IPT7*, at 1 kb and 2.1 kb upstream of *LOG1*, directly upstream of *LOG7*, and at 1.6 kb upstream of *LOG8*. **B:** *IPT7, LOG1, LOG7,* and *LOG8* expression in the fifth leaf detached from 23-day-old *p35S:AHL15-GR* plants and incubated in the dark on 5 mM MES buffer with or without 10 µM DEX for the indicated number of days*.* Expression was determined by qRT-PCR from three biological replicates (n=3), each composed of leaf material of three plants, using *ACTIN2* as the reference. The Kruskal-Wallis test was used to compare conditions, and significan*t* differences between groups are indicated by different letters (p < 0.05).

**Figure S6:** Expression of *CKX2, CKX3, CKX5,* and *ORE1* in the fifth leaf of 23-days-old wild-type (Col-0), *ahl15* loss-of-function, and *AHL15* overexpression (*AHL15 OX* lines 2 and 9) plants. Expression is relative to *ACTIN2* and was normalized using the ΔΔCT method. Error bars show the standard error of the mean; groups were compared with the Kruskal-Wallis test, and different letters indicate significan*t* differences between groups (p<0.05).

**Figure S7**. AHL15 delays the dark-induced decline in iP-type CK levels in leaves. The fifth leaf was collected from 23-day-old plants and floated on 5 mM MES with or without 10 µM DEX and incubated in the dark for the indicated number of days. Material from three leaves was pooled for each sample, and five samples were measured for each condition (n = 5). Groups were compared using the Kruskal-Wallis test, and different letters indicate significant differences between groups (p < 0.05). The iP base was not present at detectable levels.

**Figure S8.** AHL15 delays the dark-induced decline in tZ- and tZR-type CK levels in leaves. The fifth leaf was collected from 23-day-old plants and floated on 5 mM MES with or without 10 µM DEX and stored in the dark for the indicated number of days. The material of three leaves was pooled for each sample, and five samples were measured for each condition (n = 5). Groups were compared using the Kruskal-Wallis test, and different letters indicate significan*t* differences between groups (p < 0.05).

**Figure S9.** Effect of AHL15 on cZ-type CK levels in dark-incubated leaves. The fifth leaf was collected from 23-day-old plants and floated on 5 mM MES with or without 10 µM DEX and incubated in the dark for the indicated number of days. Material from three leaves was pooled for each sample, and five samples were measured for each condition (n = 5). Groups were compared using the Kruskal-Wallis test, and letters indicate significan*t* differences between groups (p < 0.05).

**Figure S10.** Effect of AHL15 on DHZ-type CK levels in dark-incubated leaves. The fifth leaf was collected from 23-day-old plants and floated on 5 mM MES with or without 10 µM DEX and incubated in the dark for the indicated number of days. For each sample, material from three leaves was pooled, and five samples were measured for each condition (n = 5). Groups were compared using the Kruskal-Wallis test, and different letters indicate significan*t* differences between groups (p < 0.05). DHZ base was not present at detectable levels in these samples.

**Figure S11.** AHL15 delays the dark-induced decline in active CK levels in leaves. The fifth leaf was collected from 23-day-old plants and floated on 5 mM MES with or without 10 µM DEX and incubated in the dark for the indicated number of days. The material of three leaves was pooled for each sample, and five samples were measured for each condition (n = 5). Groups were compared using the Kruskal-Wallis test, and different letters indicate significan*t* differences between groups (p < 0.05).

**Figure S12.** AHL15 preserves the chlorophyll content in leaves during dark incubation. **A:** Normalized Red values (Rn) based on RGB images of leaves. **B:** Total chlorophyll content determined by acetone-based chlorophyll extraction in leaf disks. For each condition in **A** and **B**, five samples were measured (n = 5), each consisting of material from the fifth leaf harvested from three individual 23-day-old p35S:AHL15-GR plants. Groups were compared with the Kruskal-Wallis test, and different letters indicate significant differences between groups (p <0.05).

**Figure S13** Trans-zeatin or CKX inhibitor treatment decreases dark-induced senescence in Col-0 and *ahl15* loss-of-function leaves. The fifth leaf of 23-day-old plants was harvested and incubated in the dark for 5 days on 5 mM MES solution with or without 1 uM hormone or CKX-i . **A:** Normalized Red values (Rn) based on RGB images of leaves. **B:** Total chlorophyll content determined by acetone-based chlorophyll extraction. Grey dots indicate individual leaves. Groups were compared with the Kruskal-Wallis test (n = 10 for each condition), and different letters indicate significan*t* differences between groups (p<0.05).

**Table S1:** Primers used for RT-qPCR.

| **Name** | **Sequence (5' to 3')** | **function** |
| --- | --- | --- |
| ACTIN2-q-fw | TTCCTCAGCACATTCCAGCAGAT | reference gene for RT-qPCR |
| ACTIN2-q-rev | AACGATTCCTGGACCTGCCTCATC | reference gene for RT-qPCR |
| AHL15-q-fw | CTGAGCGGTAGTGGTTTGGT | RT-qPCR of AHL15 |
| AHL15-q-rev | AGGAGAGCCAGACGTAGGAA | RT-qPCR of AHL15 |
| ORE1-q-fw | CTTACCATGGAAGGCTAAGATGGG | RT-qPCR of ORE1 |
| ORE1-q-rev | TTCCAATAACCGGCTTCTGTCG | RT-qPCR of ORE1 |
| SAG12-q-fw | ACCGGGACATCCTCATAACCTG | RT-qPCR of SAG12 |
| SAG12-q-rev | ACAAAGGCGAAGACGCTACTTG | RT-qPCR of SAG12 |
| SAG13-q-fw | AGGGAGCATCGTGCTCATATCC | RT-qPCR of SAG13 |
| SAG13-q-rev | CCAGCTGATTCATGGCTCCTTTG | RT-qPCR of SAG13 |
| SEN1-q-fw | GTTGTCGTTGCTTTCCTCCATC | RT-qPCR of SEN1 |
| SEN1-q-rev | GTCATCGGCTATTTCTCCACCT | RT-qPCR of SEN1 |
| CKX2-q-fw | GAGGTTTGGGTCAATTTGGA | RT-qPCR of CKX2 |
| CKX2-q-rev | AGAGCATCCGAAACCATTTG | RT-qPCR of CKX2 |
| CKX3-q-fw | TCTCAATACACAGTCAACGAGGA | RT-qPCR of CKX3 |
| CKX3-q-rev | TCGTACATAAACCCTCTTACATGG | RT-qPCR of CKX3 |
| CKX5-q-fw | CCATGGTCCTCAAATTAGTAACG | RT-qPCR of CKX5 |
| CKX5-q-rev | TCTGAGCATCTCATCACCTCTC | RT-qPCR of CKX5 |
| LOG1-q-fw | TTTTGTGGAAGTAGTGCTGGTAATA | RT-qPCR of LOG1 |
| LOG1-q-ref | AAATCAAACCCATTAAACCAAT | RT-qPCR of LOG1 |
| LOG2-q-fw | ACACTAAACCGGTGGGACTATTGAA | RT-qPCR of LOG2 |
| LOG2-q-rev | GGAGCGTTTGGAGCCGAGAC | RT-qPCR of LOG2 |
| LOG7-q-fw | CGCTGTTAACGTTTATTGATAAGGC | RT-qPCR of LOG7 |
| LOG7-q-rev | TTATCTCATCAAACTCCGGTTCA | RT-qPCR of LOG7 |
| LOG8-q-fw | GTTTATGGAGGAGGAAGTGTTGGAT | RT-qPCR of LOG8 |
| LOG8-q-rev | TCACCAGATATCTCAATTGGCAT | RT-qPCR of LOG8 |
| IPT7-q-fw | ACCTAACGGCCACCCAGTAT | RT-qPCR of IPT7 |
| IPT7-q-rev | CGGAGTAATTCGCTTTTGGA | RT-qPCR of IPT7 |
|  | * fw: forward, rev: reverse |  |
